# Early screening outcomes among non-immigrants and immigrants targeted by BreastScreen Norway, 2010–2019

**DOI:** 10.1177/14034948221078701

**Published:** 2022-03-31

**Authors:** Jonas E. Thy, Sameer Bhargava, Marthe Larsen, Lars A. Akslen, Solveig Hofvind

**Affiliations:** 1 Cancer Registry of Norway, Norway; 2 Division of Oncology, Department of Medicine, Bærum Hospital, Vestre Viken Hospital Trust, Norway; 3 Centre for Cancer Biomarkers CCBIO, Department of Clinical Medicine, Section for Pathology, University of Bergen, Norway; 4 Department of Pathology, Haukeland University Hospital, Norway; 5 Department of Health and Care Sciences, UiT The Arctic University of Norway, Norway

**Keywords:** Mass screening, mammography, breast cancer, immigrants, early detection of cancer, participation, early performance measures

## Abstract

**Aims:**

This study aimed to analyse results on early screening outcomes, including recall and cancer rates, and histopathological tumour characteristics among non-immigrants and immigrants invited to BreastScreen Norway.

**Methods:**

We included information about 2, 763,230 invitations and 2,087,222 screening examinations from 805,543 women aged 50–69 years who were invited to BreastScreen Norway between 2010 and 2019. Women were stratified into three groups based on their birth country: non-immigrants, immigrants born in Western countries and immigrants born in non-Western countries. Age-adjusted regression models were used to analyse early screening outcomes. A random intercept effect was included in models where women underwent several screening examinations.

**Results:**

The overall attendance was 77.5% for non-immigrants, 68% for immigrants from Western countries and 51.5% for immigrants from non-Western countries. The rate of screen-detected cancers was 5.9/1000 screening examinations for non-immigrants, 6.3/1000 for immigrants from Western countries and 5.1/1000 for immigrants from non-Western countries. Adjusted for age, the rate did not differ statistically between the groups (*p*=0.091). The interval cancer rate was 1.7/1000 screening examinations for non-immigrants, 2.4/1000 for immigrants from Western countries and 1.6/1000 for non-Western countries (*p*<0.001). Histological grade was less favourable for screen-detected cancers, and subtype was less favourable for interval cancers among immigrants from non-Western countries versus non-immigrants.

**Conclusions:**

**There were no differences in age-adjusted rate of screen-detected cancer among non-immigrants and immigrants from Western countries or non-Western countries among women attending BreastScreen Norway between 2010 and 2019. Small but clinically relevant differences in histopathological tumour characteristics were observed between the three groups.**

## Introduction

Breast cancer incidence and mortality vary across the world [1]. The highest incidence is reported for women in Northern America, Western Europe, Australia and New Zealand [1]. In Norway, subpopulations of immigrants from non-Western countries appear to have lower incidence than non-immigrants do, but they also appear to have less favourable tumour characteristics and lower survival [2–4].

The European Commission Initiative on Breast Cancer (ECIBC) and the International Agency for Research on Cancer both recommend mammographic screening for asymptomatic women aged 50–69 years [5,6]. The ECIBC recommends organised mammographic screening over no screening, and it considers the benefits to outweigh the harms. To attain these benefits, early outcome measures, such as attendance, cancer detection and distribution of histopathological tumour characteristics, should reach certain thresholds [7]. For example, an attendance rate of ⩾75% is considered desirable [7]. Such thresholds do not differentiate between immigrants and non-immigrants.

In keeping with these recommendations, BreastScreen Norway offers all women 50–69 years of age mammographic screening every two years. During 1996–2015, the attendance rate was 56% for immigrants and 78% for non-immigrants [8]. Additionally, among immigrants, recall rate was higher, screen-detected cancer lower and histopathological tumour characteristics less favourable among immigrants compared to non-immigrants. The latter finding is in keeping with research demonstrating that women with screen-detected cancers have more favourable histopathological characteristics than women with interval- or clinically detected cancers [9,10].

Migration between countries is increasing globally, with hundreds of millions of people living outside their country of origin. Since 1970, immigration to Norway has exceeded emigration, and 15% of the current population was born outside Norway [11]. The immigrant population has changed over time. The proportion of immigrants in Norway born in Eastern Europe, Asia or Africa has increased since 1970 and currently makes up the majority of the immigrant population [11]. Further, although family immigration was the most common reason for immigration until the expansion of the European Union in 2004, the most common reason since then has been labour immigration [11]. Updated and additional knowledge is needed to optimise organised screening for breast cancer in Norway and in other countries because of changes in the immigrant population.

To fill some of the knowledge gaps related to immigrants and mammographic screening, we took advantage of data available from the Cancer Registry of Norway and compared results of early screening outcomes among non-immigrants and immigrants targeted by BreastScreen Norway between 2010 and 2019. We defined early screening outcomes as attendance, recall, cancer detection (screen-detected and interval cancer), as well as surgical treatment and histopathological tumour characteristics (histopathological type, tumour diameter, histological grade, lymph node status and subtypes).

## Methods

We received de-identified data from the Cancer Registry of Norway, which administers BreastScreen Norway. The de-identified data contained an encrypted identifier, and the individuals could not be identified directly. Data completeness at the Cancer Registry of Norway is almost 100% for solid tumours [12]. The data protection officer for research at Oslo University Hospital approved our study (2020/12601).

BreastScreen Norway started in 1996 and was nationwide by 2005. Invitation to screening includes a scheduled time and place for examination and an information leaflet about the screening procedure and the potential benefits and harms of mammographic screening. The invitation letter and information leaflet are sent electronically (digital mail) or by postal service. Digital mail was implemented in Norway in 2016 and is used for invitations to BreastScreen Norway if activated by the invitee [9]. The invitation letter and information leaflet are written in Norwegian, but the invitation refers women to a website with information in English, Somali, Urdu, Polish and Arabic.

A reminder is sent to non-attending women four to six weeks after the originally scheduled appointment. These women can call their regional breast centre to schedule a new appointment. All women have to pay a user fee of €26 for each screening examination.

Screening is conducted at 24 stationary and four mobile units across the country. BreastScreen Norway performs independent double reading with consensus. Screen reading, further assessment, treatment and follow-up take place at 17 breast centres primarily located at regional or university hospitals.

### Study populations I and II

**Figure 1.**
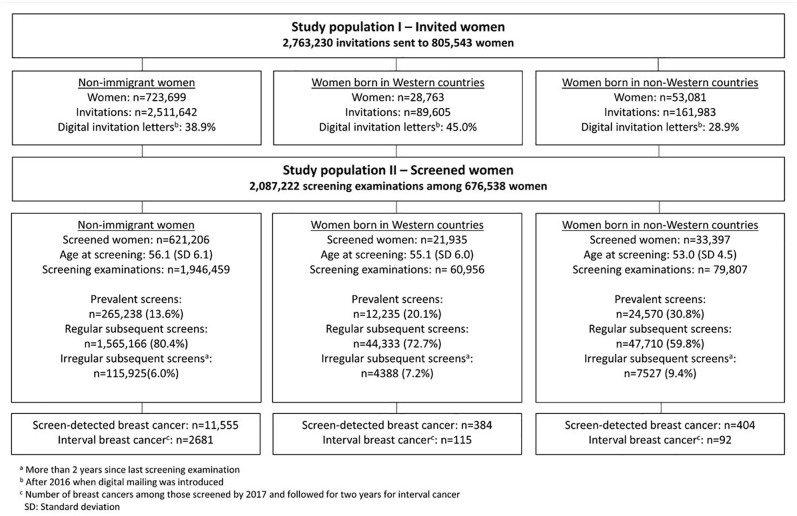
Study populations I and II – number of invitations, screening examinations and women, age at screening, number of prevalent, regular subsequent and irregular subsequent screening examinations and number of screening-detected and interval cancers (2010-2019).

We received information about 2,852,877 invitations sent to 832,596 women invited to BreastScreen Norway during the study period, January 2010 to December 2019, and grouped these into study populations I and II. In both populations, we excluded invitations sent to women after a breast cancer diagnosis (*N*=61,776) and women with no information about country of birth (7838 women and 27,871 invitations). Study population I included 2,763,230 invitations and 805,543 women (Figure 1). Of these, 10.2% were immigrants.

Study population II included the 84% of invited women in study population I who attended at least once. Of these, 8.2% were immigrants. Women in this study population underwent 2,087,222 screening examinations (Figure 1).

### Birth countries

Information about birth country has been available for all women in the Cancer Registry databases since 2018 and was used to classify women into one of three ‘birth country groups’: non-immigrants, immigrants from Western countries and immigrants from non-Western countries [13]. We classified the women according to their country of birth. All women born in Norway were classified as non-immigrants.

Immigrants were classified into Western and non-Western countries to reflect differences in breast cancer incidence in their countries of birth [1]. Western countries included countries in Western Europe, Northern America, Australia and New Zealand, while non-Western countries included all other countries (Supplemental Table I).

### Measuring early screening outcomes

Attendance rate was defined as the number of screening examinations divided by the number of invitations during the study period. We defined prevalent screening examinations as the first screening examination within the programme, while subsequent screening examinations were defined as a consecutive screening examination in the programme. Subsequent examinations were classified as regular or irregular, where regular attendance was that occurring <30.5 months after the previous examination and irregular attendance occurred ⩾30.5 months after the previous examination.

Knowing the screening history among women might help us in the interpretation of the results, as the rate of screen-detected cancers are usually higher and the tumour characteristics less favourable for prevalent versus subsequently screened women.

The recall rate was defined as the number of screening examinations leading to further assessment due to abnormal mammographic findings divided by the total number of screening examinations. Screen-detected cancer was defined as breast cancer diagnosed after a recall and within six months after the screening examination. We included ductal carcinoma in situ (DCIS) and invasive cancer in our definition of breast cancer. The screen-detected cancer rate was calculated as the number of cancer cases divided by the total number of screening examinations. Positive predictive values (PPV) were estimated as the percentage of screen-detected cancer among all recalled women (PPV-1) and among all biopsied women (PPV-3). We defined interval cancer as breast cancer diagnosed within 24 months of a negative screening result or within 6–24 months of a false-positive screening examination [9].

Histopathological type was classified as DCIS, invasive ductal carcinoma of no special type, invasive lobular carcinoma or other invasive cancers [14]. For invasive cancers, we presented tumour diameter (largest focus in cases with multifocal disease), histological grade, lymph node status and molecular subtypes defined using immunohistochemical (IHC) surrogate markers. Using oestrogen, progesterone and human epidermal growth factor receptor 2 (Her2) status, we divided the tumours into luminal A-like, luminal B-like (Her2–), luminal B-like (Her2+), Her2+ (non-luminal) and triple negative based on a modification of the St Gallen guidelines (without Ki67) [15].

### Statistical analysis

We used study population I to describe attendance rates, while study population II was used to describe recall rates, PPV, biopsy rates and cancer rates. We presented frequencies and proportions for categorical variables. Age was described using means and standard deviations, and tumour diameter was described using medians and interquartile ranges due to right-skewed distribution. Attendance rates, recall rates, PPV and cancer detection rates were calculated as described in the definitions above. Attendance rates were calculated separately for digital and postal invitations.

All other early outcome measures were analysed using mixed models because of non-independence between screening examinations. The exposure of interest was birth country group (non-immigrants, Western and non-Western). The identifier for each woman was added as a random effect. Tumour characteristics for invasive tumours were analysed with linear regression or binary, ordinal or multinomial logistic regression, depending on the outcome variable. All models were adjusted for age. We used Stata v16.1 for Windows (StataCorp, College Station, TX) for all statistical analyses.

## Results

The 723,699 non-immigrant women in our study received 2,511,642 invitations, while the 28,763 immigrant women from Western countries received 89,605 invitations and the 53,081 immigrant women from non-Western received 161,983 invitations (Figure 1). On average, these women received 3.5, 3.1 and 3.1 invitations during the study period, respectively.

Among non-immigrants, 85.8% (621,206/723,699) of the women attended BreastScreen Norway at least once compared to 76.3% (21,935/28,763) of the immigrants from Western countries and 62.9% (33,397/53,081) of the immigrants from non-Western countries (Figure 1). The proportion of prevalent examinations was 13.6% for non-immigrants, 20.1% for immigrants from Western countries and 30.8% for immigrants from non-Western countries.

**Figure 2.**
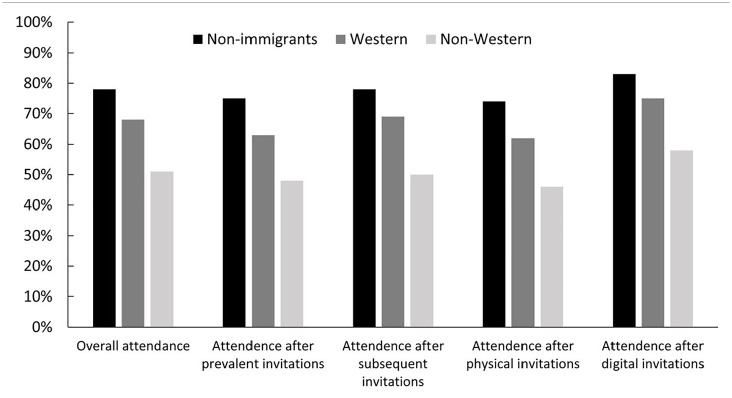
Attendance rates (%) in BreastScreen Norway between 2010 and 2019 among non-immigrants and immigrants from Western and non-Western countries, stratified by screening history (prevalent and subsequent invitation) and invitation type (physical or digital).

The overall attendance rate was 77.5% for non-immigrants, 68% for immigrants from Western countries and 51.5% for immigrants from non-Western countries (Figure 2). Postal invitations resulted in attendance rates of 74.8%, 63.2% and 47.5%, while digital invitations resulted in attendance rates of 82.9%, 75.3% and 58.5%, respectively.

Recall and biopsy rates were significantly lower among non-immigrants compared to immigrants from both Western and non-Western countries (*p*<0.001; Table I). Similar results were observed for prevalent and regular subsequent screening examinations (Supplemental Table II). The rate of screen-detected cancer was 0.59% for non-immigrants, 0.63% for immigrants from Western and 0.51% for immigrants from non-Western countries (Table I). These rates differed substantially between geographical regions (Supplemental Table III). After adjusting for age, birth country group was not associated with screen-detected cancer rates (*p*=0.091; Table I). For regular subsequent examinations, screen-detected cancer rates were significantly higher among non-immigrants compared to immigrants from non-Western countries (*p*=0.026; Supplemental Table II). PPV-1 was 17.6% and PPV-3 was 41% for non-immigrants, while it was 15.4% and 36.4% for immigrants from Western countries and 11.6% and 26.4% for immigrants from non-Western countries, respectively (Table I). Adjusted for age, birth country group was associated with PPV-1 and PPV-3 (*p*<0.001). The interval cancer rate was 0.17% for non-immigrants, 0.24% for immigrants from Western countries and 0.16% for immigrants from non-Western countries. This difference was statistically significant after adjusting for age. Surgical procedure did not differ statistically between groups (*p*=0.280).

**Table I.** Number of screening examinations performed in BreastScreen Norway between 2010 and 2019.

Screening examinations	All women (*N*=2,087,222)		Non-immigrants (*N*=1,946,459)		Western (*N*=60,956)		Non-Western (*N*=79,807)		*p*-Value^b^
	*n*	%	*N*	%	*N*	%	*n*	%	
Recall	71,549	3.4	65,561	3.4	2498	4.1	3490	4.4	<0.001
Biopsy	30.753	1.5	28,169	1.5	1056	1.7	1528	1.9	<0.001
Screen-detected cancers	12,343	0.59	11,555	0.59	384	0.63	404	0.51	0.091
Surgical procedure									0.587
Mastectomy	2528	21.4	2350	21.2	87	23.6	91	24.3	
BCT	9216	78.6	8654	78.8	277	76.4	285	75.7	
Information not available	113		99		2		12		
PPV-1	12,343/71,549	17.3	11,555/65,561	17.6	384/2498	15.4	404/3490	11.6	<0.001
PPV-3	12,343/30,753	40.1	11,555/28,169	41.0	384/1056	36.4	404/1528	26.4	<0.001
Interval cancers^a^	2888	0.18	2681	0.17	115	0.24	92	0.16	0.001
Surgical procedure									0.280
Mastectomy	1070	39.2	987	38.9	40	39.1	43	48.8	
Breast conserving therapy	1654	60.8	1543	61.1	64	60.9	47	51.2	
Information not available	91		84		6		1		

Number and rates of recall, biopsy, screen-detected and interval cancer, positive predictive value of recalls (PPV-1) and biopsies (PPV-3) and surgical procedures for non-immigrants and immigrants from Western and non-Western countries are shown.

a Calculated based on 1,647,270 screening examinations between 2010 to 2017.

b Overall *p*-value for birth country group calculated from age-adjusted regression.

### Histopathological tumour characteristics

Invasive tumours represented 82.1% of the screen-detected cancers among non-immigrants, 78.4% among immigrants from Western countries and 82.4% among immigrants from non-Western countries (Table II). Invasive tumours represented 93.6% of the interval cancers among non-immigrants, 93% among immigrants from Western countries and 94.6% among immigrants from non-Western countries (Table III). Adjusted for age, histological grade was associated with birth country group (*p*=0.001) for screen-detected cancers. Further, molecular subtype was associated with birth country group (*p*=0.020) for interval cancers. Tumours were more likely to be luminal-A-like among non-immigrants versus immigrants from Western countries, and less likely to be triple negative among non-immigrants versus immigrants from non-Western countries (Table III).

**Table II.** Histopathological tumour characteristics for screen-detected cancers among non-immigrants and immigrants from Western and non-Western countries.

	All women		Non-immigrants		Western		Non-Western		*p*-Value^a^
Age at diagnosis (years), *M* (*SD*)	60.0 (6.0)		60.2 (5.9)		59.5 (6.4)		56.9 (5.4)		<0.001
All tumours, *n*	12,343		11,555		384		404		
Histological type, *n*, %									
Ductal carcinoma in situ	2217	18.0	2063	17.9	83	21.6	71	17.6	0.214
Invasive ductal carcinoma NST	8561	69.4	8038	69.6	245	63.8	278	68.8	
Invasive lobular carcinoma	1077	8.7	999	8.7	42	10.9	36	8.9	
Other invasive	488	4.0	455	3.9	14	3.7	19	4.7	
Invasive cancers, *n*	10,126		9492		301		333		
Tumour diameter, median (IQR)	13 (9–19)		13 (9–19)		14 (8–20)		13 (9–21)		0.322
Histological grade, *n*, %									0.001
1	2946	29.5	2756	29.5	107	36.7	83	24.3	
2	4876	48.7	4594	48.9	135	46.3	147	46.9	
3	2175	21.8	2028	21.7	52	17.1	95	28.8	
Information not available, *n*	129		114		7		8		
Lymph node status, *n*, %									0.941
Positive	2055	20.3	1928	20.2	55	19.1	72	22.7	
Information not available	218		199		10		9		
Immunohistochemical subtype, *n*, %									0.472
Luminal A-like	5962	61.6	5596	61.5	181	66.5	185	60.7	
Luminal B-like (Her2–)	1259	13.0	1188	13.1	36	13.2	35	11.5	
Luminal B-like (Her2+)	1652	17.1	1553	17.1	42	15.4	57	18.7	
Her2+ (non-luminal)	302	3.1	283	3.1	7	2.6	12	3.9	
Triple negative	508	5.3	486	5.3	6	2.2	16	5.3	
Information not available	446		418		16		12		

a Overall *p*-value for birth country group calculated from age-adjusted regression.

*SD*: standard deviation; IQR: interquartile range; NST: no special type; Her2: human epidermal growth factor receptor 2.

**Table III.** Histopathological tumour characteristics for interval cancers among non-immigrants and immigrants from Western and non-Western countries.

	All women		Non-immigrants		Western		Non-Western		*p*-Value^a^
Age at diagnosis (years), *M* (*SD*)	60.4 (6.0)		60.5 (6.0)		59.5 (5.8)		58.3 (5.2)		<0.001
All tumours, *n*	2881		2681		115		92		
Histological type, *n*, %									0.635
Ductal carcinoma in situ	184	6.4	171	6.4	8	7.0	5	5.4	
Invasive ductal carcinoma NST	2223	77.0	2056	76.7	90	78.3	77	83.7	
Invasive lobular carcinoma	378	13.1	355	13.2	16	13.9	7	7.6	
Other invasive	103	3.6	99	3.7	1	0.9	3	3.3	
Invasive cancers, *n*	2704		2510		107		87		
Tumour diameter, median (IQR)	18 (12–25)		18 (12–25)		18 (12–25)		17 (11–26)		0.267
Histological grade, *n*, %									0.517
1	394	14.9	368	15.0	16	15.2	10	13.2	
2	1194	46.1	1100	45.9	57	52.2	37	44.7	
3	1015	39.0	947	39.1	31	32.6	37	42.1	
Information not available	101		95		3		3		
Lymph node status, *n*, %									0.758
Positive	925	34.2	861	34.0	35	36.8	29	36.7	
Information not available	138		125		9		4		
Immunohistochemical subtype, *n*, %									0.020
Luminal A-like	1237	49.2	1169	49.7	33	37.1	35	46.7	
Luminal B-like (Her2–)	327	13.0	300	12.8	19	21.4	8	10.7	
Luminal B-like (Her2+)	473	18.8	447	19.0	16	18.0	10	13.3	
Her2+ (non-luminal)	172	6.8	156	6.6	11	12.4	5	6.7	
Triple negative	308	12.2	281	11.9	10	11.2	17	22.7	
Information not available	186		176		6		4		

a Overall *p*-value for birth country group calculated from age-adjusted regression.

## Discussion

In this study of 805,543 women invited to BreastScreen Norway between 2010 and 2019, we observed that immigrants had lower attendance than non-immigrants. We observed higher attendance rates among women who received digital versus postal invitation letters in all three birth country groups. After adjusting for age at diagnosis, the rate of screen-detected cancer did not differ between the three groups, while the rate of interval cancer was higher among immigrants from non-Western countries. Small but clinically relevant differences in histopathological tumour characteristics were observed between the three groups.

Our finding of lower attendance among immigrants versus non-immigrants has also been reported in other studies [16]. These findings might be partly attributed to lower socio-economic status and less use of digital postal services among immigrants. In our study, we observed a higher attendance rate among women invited after digital versus postal invitation within all three birth country groups. A recent report from the Norwegian Directorate of Health suggested that elderly people with less formal education and those with chronic illnesses are less able to adopt digital health services [17]. Women who have opted for digital invitations may thus have higher socio-economic status, which may partly explain why they have higher attendance rates than women who receive physical invitations. Indeed, we have previously shown that Norwegian women with higher socio-economic status are more likely to attend BreastScreen Norway than women with lower socio-economic status [18].

Lower recall and biopsy rates among non-immigrant women could be explained by a higher proportion of subsequent screening examinations and thus prior mammograms for comparison in the interpretation compared to immigrant women. Ethnic variations in mammographic density might have also influenced these results [19].

Our previous results from 1996 to 2015 showed that the rate of screen-detected cancer was higher among immigrants from Western countries and lower among those from non-Western countries compared to non-immigrants [8]. We found similar results in our study, but they were no longer statistically significant after adjusting for age. Similar rates of screen-detected cancer after adjusting for age could signal higher socio-economic status among attending immigrants, reflecting the healthy migrant effect [20]. Further, the current study included at least two additional screening rounds, resulting in an older immigrant population with a longer history in the country and a higher proportion of subsequent screening examinations. Since our previous study was published, immigrant health has been a focus of attention in politics, the media and research [2,4,8,16,21,22].

In keeping with our results from 1996 to 2015, interval cancers in this study were more likely to be triple negative among immigrants from non-Western countries versus non-immigrants. Triple-negative breast cancer is associated with lower survival than other subtypes [23]. Additionally, in the current study, interval cancers were less likely to be luminal-A-like among immigrants from Western countries than among non-immigrants. A Belgian study found that Arab/Moroccan women had a lower proportion of luminal A-like tumours and a higher proportion of luminal B-like tumours than European women [24]. Luminal A-like tumours are associated with a more favourable prognosis than other subtypes [23]. These findings need replication but could indicate that there are differences in molecular subtypes at diagnosis among women from different parts of the world.

Breast cancer is the most common cancer among women worldwide. However, obtaining sufficient power when analysing histopathological tumour characteristics among immigrants is challenging because of sparsity in subgroups. We could have extended our study period to include data as far back as 1996, but during 1996–2020, there were demographic changes in the immigrant populations and changes to the screening programme [9,11]. Further, results generated from data obtained 20–25 years ago may have limited value today. Nordic collaboration through the Nordic Cancer Union offers an alternative opportunity to increase sample size. The Nordic countries have similarly organised health services and high-quality cancer registries with screening data. However, results are difficult to compare due to differences in the organisation of screening programmes [25] and the composition of immigrant populations.

Both immigrants and non-immigrants may face challenges with respect to health literacy, but immigrant women may face additional challenges because invitations to screening are provided in Norwegian. The Norwegian language is used by few outside of Norway, and immigrants may benefit from translated information [26]. However, the COVID-19 pandemic has shown that it is not sufficient to translate information on health awareness and health behaviour, and this might also be relevant in the context of mammographic screening. To be useful, health information must first reach the recipient and then be read, understood and absorbed. Additionally, illiterate women may need oral information. In a study of Pakistani women in Norway, we showed that some women prefer information about screening through their family members and general practitioners rather than from governing institutes [27]. This finding might also be true for immigrants from other countries.

We used detailed, high-quality, registry-based data with nationwide coverage, where all data, including screening history and tumour characteristics, were linked on an individual level. A major limitation in this study, however, is that immigrants were classified into two large groups. This may hide important information for women from different countries. Previously, we have shown major differences in attendance rates even between women born in neighbouring countries [18]. Our somewhat crude classification may also hide relevant differences in conceptions of health, understanding of cancer, religiosity and pre- and post-migratory factors, which could have affected our results. Nonetheless, we determined that a more precise division was not appropriate due to a lack of statistical power.

Further limitations are present in our study, including missing data. For example, we did not have information about mammographic density or baseline tumour diameter for women with locally advanced breast cancer who underwent neoadjuvant chemotherapy. We do not know whether the resulting underestimation in tumour diameter equally affected our study groups. We also lacked information about multifocality and multicentricity. However, data quality at the Cancer Registry of Norway is high, and the proportion of missing data in our study was relatively low [12]. Our classification into molecular subtypes did not include Ki67 proliferation status because this information was not routinely collected before 2012. This marker may be relevant to include in future studies because it is relevant for both choice of treatment through molecular classification and prognostic estimation. Other markers may also be identified as relevant in future studies. For example, since immunotherapy has been included in the first-line treatment of metastatic triple-negative breast cancers that express PD-L1 [28], PD-L1 expression may also be important in future studies.

In conclusion, results from BreastScreen Norway showed lower attendance rates among immigrants compared to non-immigrants between 2010 and 2019. We found no statistically significant differences in age-adjusted rate of screen-detected cancer. Small but clinically relevant differences in histopathological tumour characteristics were observed between the three groups, with less favourable characteristics among immigrants from non-Western countries. The results of our study suggest that additional attention and resources are needed to improve health equity between non-immigrants and immigrants in Norway.

## Supplemental Material

sj-xlsx-1-sjp-10.1177_14034948221078701 – Supplemental material for Early screening outcomes among non-immigrants and immigrants targeted by BreastScreen Norway, 2010–2019 Click here for additional data file. Supplemental material, sj-xlsx-1-sjp-10.1177_14034948221078701 for Early screening outcomes among non-immigrants and immigrants targeted by BreastScreen Norway, 2010–2019 by Jonas E. Thy, Sameer Bhargava, Marthe Larsen, Lars A. Akslen and Solveig Hofvind in Scandinavian Journal of Public Health
